# Supplementary material for: Merging public elementary schools to reduce racial/ethnic segregation
Source: PNAS Nexus. 2025 Mar 4;4(3):pgaf050. doi: 10.1093/pnasnexus/pgaf050 (PMC11879517; doi:10.1093/pnasnexus/pgaf050)
Supplement: pgaf050_Supplementary_Data [file pgaf050_supplementary_data.pdf]

Supplementary Materials for "Merging public elementary schools to reduce racial/ethnic segregation"

Madison Landry and Nabeel Gillani
Plural Connections Group
Northeastern University
Boston, MA USA
{landry.ma, n.gillani}@northeastern.edu

Contents

S1 Sensitivity analyses **3**
S1.a Optimizing for Black/Hispanic and White/Asian integration 3
S1.b Opt-outs 3
S1.c School enrollment minimum constraint 5
S1.c.a School closures 8
S1.d Inter-district mergers 8

S2 Data and code release **9**

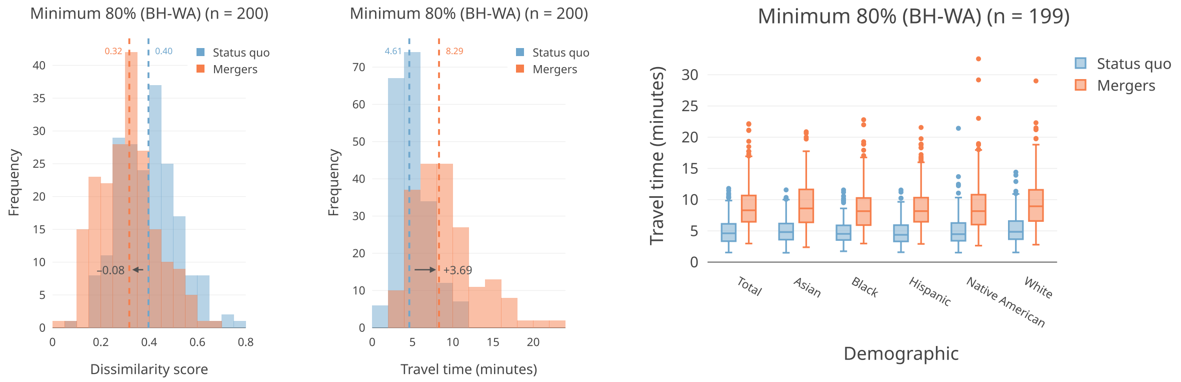

(a) Dissimilarity score (b) Travel times (c) Travel times across demographic

Figure S1: Impact on integration results when optimizing for Black/Hispanic and White/Asian integration.

S1 Sensitivity analyses

To assess the variability & generalizability of our results across different design choices of the constraint programming problem, we conduct four sensitivity analyses.

1. *Optimizing for Black/Hispanic and White/Asian integration*: The main text reports results for optimizing for White and non-White integration. How may impacts on integration differ if optimizing for Black/Hispanic and White/Asian integration?

2. *Opt-outs*: How may impacts on integration differ, considering a subset of families involved in a school merger may opt out of their assigned schools?

3. *School enrollment minimum constraint*: We set the minimum school capacity constraints to 0.8 (see Equation 7 in the main text). How may impacts on integration differ, considering other minimum school enrollment thresholds: 0.9, 0.7, and 0.0?

4. *Interdistrict*: How may impacts on integration differ, if mergers across district lines are allowed?

S1.a Optimizing for Black/Hispanic and White/Asian integration

Fig. S1 summarizes the results for optimizing Black/Hispanic and White/Asian integration, defined using the same dissimilarity index as presented in the main text, except now with students classified as either Black/Hispanic or White/Asian (instead of White or non-White—students who don't fit into Black/Hispanic or White/Asian groups are not included in the simulations). Results are nearly identical to optimizing for White and non-White integration: the dissimilarity score decreases by a median relative 20.% across districts in our sample, and median travel times increase by just under 4 minutes. Fig. S2 plots the dissimilarity results for this sensitivity analysis against those presented in the main text (optimizing for White/non-White integration), with some outlier districts marked. Some districts such as Sacramento City in California have greater potential for integration when measured across Black/Hispanic and White/Asian groups, whereas other districts such as Howard County in Maryland have greater potential for integration when measured across White and non-White groups. In general, however, there is a strong correlation between both sets of expected outcomes across districts.

S1.b Opt-outs

Anticipating rates of family opt-out in the face of integration policies is a challenging task. Similar to (3), we develop a basic model (based on demographic-specific rates of choice into magnet

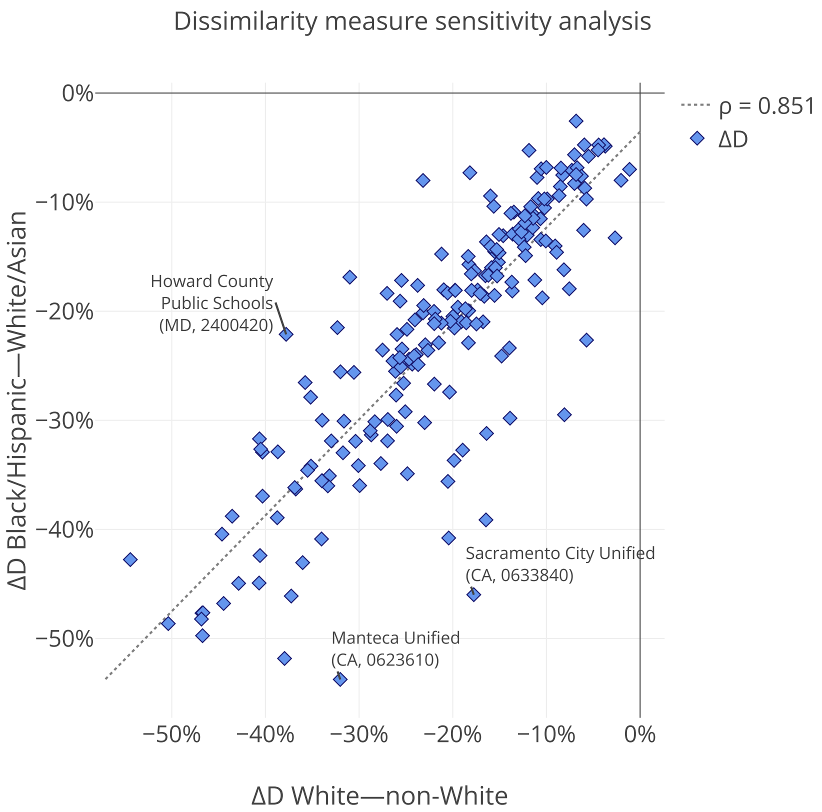

Figure S2: Resulting changes in dissimilarity, when optimizing for Black/Hispanic and White/Asian integration, versus when optimizing for White and non-White integration. ($\rho = 0.851$, $p < 10^{-56}$; $r = 0.860$, $p < 10^{-59}$)

and charter schools within districts' boundaries) to estimate how much families' decisions to leave schools post pairing/tripling might undermine desegregation. We use the 2021/2022 NCES Common Core of Data to identify elementary charter and in-district magnet schools located within at least one of the elementary school attendance boundaries across the 98 districts in our sample. Across the 200 districts in our sample, there are 1,848 such schools located in 150 districts. We estimate that the median ratio of elementary students per district attending a charter or magnet school compared to a closed-enrollment boundary-based school is approximately just over 13%, with similar rates of uptake (on average) for White students and students of color.

For each district, we compute the ratio of White students and students of color, respectively, who attend a charter or magnet school compared to a closed-enrollment elementary school with attendance boundaries. To estimate how selecting charter or magnet schools might impact the extent to which mergers foster integration, we assume that students involved in a merger opt out of their schools post-merger at a rate that reflects the existing rate of charter/magnet uptake for their racial/ethnic group. For example, imagine a district with 100 White students and 100 students of color, where the ratio of White students and students of color who opt for charter/magnet options is 0.2 and 0.1, respectively. Next, imagine that after running our algorithm, 50 White students and 30 students of color would be involved in a merger. Applying our opt-out model would yield $50 \times 0.2 = 10$ White students and $30 \times 0.1 = 3$ students of color leaving the district, respectively. We then re-compute the dissimilarity index for the district assuming these students are no longer attending district schools to arrive at an expected level of segregation after factoring in opt-outs. Doing this across the districts in our sample yields a median relative decrease in dissimilarity of 17%, which is marginally lower than the 20% suggested by a model that does not factor in opt-outs. This suggests that while school choice options may attenuate the impacts school mergers have on integration, choice is unlikely to completely undo these impacts.

This is, of course, a simplified model of family choice. It does not account for private or other out-of-district options; nor does it factor in other practical constraints like the capacities of charter and magnet programs, which may limit the extent to which families can opt into them. In general, estimating how families might select schools is difficult, even in the face of deep data access from districts (5). Our results echo other findings analyzing the impacts that integrative student assignment policies might have, most notably (4), which suggest that families are likely to respond to such policies in ways that hinder their overarching objectives, but that even in the face of these responses, such policies can still help foster progress towards more diverse and integrated schools.

S1.c School enrollment minimum constraint

We report on results of three other minimum enrollment constraints: a more restrictive constraint (0.9), a less restrictive constraint (0.7), and no minimum school enrollment (0.0). The latter extreme provides a more complete understanding of the variability and allows for discussion on potential school closures that might emerge from pairings and triplings. Primary results are summarized in Figs. S3,S4.

By loosening the minimum school capacity constraint, a greater number of schools may be clustered. In particular, fractions of 0.9, 0.8, 0.7, and 0.0 resulted in, respectively, 4,774, 6,108, 6,256, and 7,116 clustered schools across the top 200 districts by population. The magnitude of change in dissimilarity score likewise improves, with relative decreases of, respectively, 14.6%, 19.5%, 22.5%, and 29.3% (Fig. S3a–d). Median changes in travel time for switching students tend to stay relatively stable, under 4 minutes (Fig. S3e–h).

In terms of relative change in school enrollment, all simulations had a mode of 0.00, with right-skewed distributions for tighter enrollment constraints and a more symmetric distribution for no

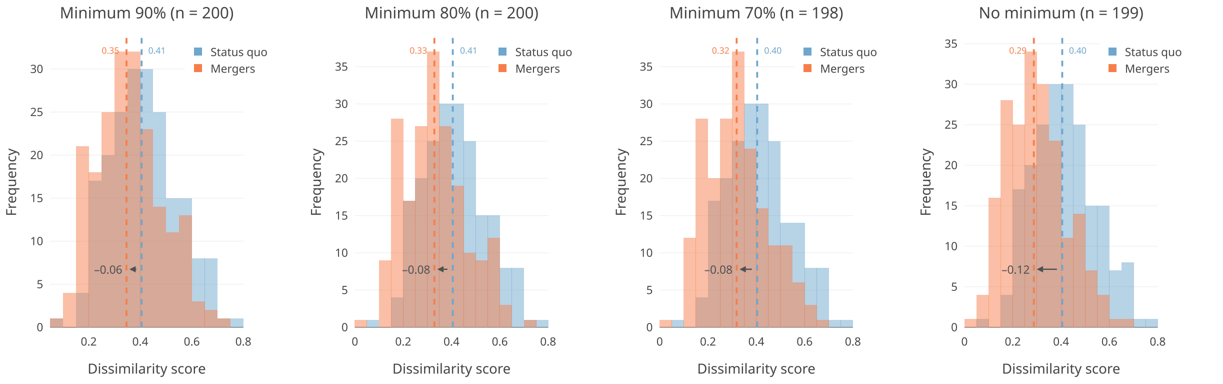

(a) Minimum 90% (b) Minimum 80% (c) Minimum 70% (d) No minimum

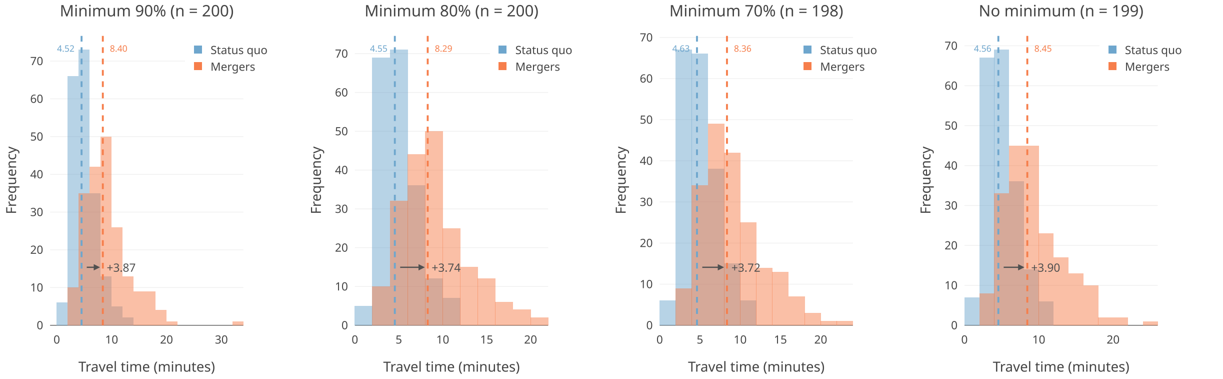

(e) Minimum 90% (f) Minimum 80% (g) Minimum 70% (h) No minimum

Figure S3: Histogram plots summarizing the results of the minimum enrollment constraint sensitivity analyses, loosening constraints from left to right. (a)–(d) Impacts on integration by change in dissimilarity score, 20 bins; and (e)–(h) respective changes in car travel times for students who would be switching schools, 20 bins. Sample sizes of less than 200 are due to the failure of the algorithm to find feasible mergers.

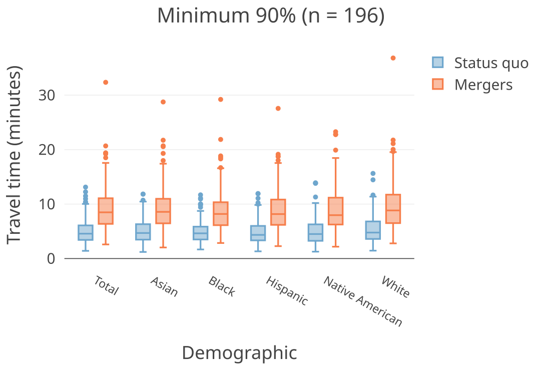

(a) Minimum 90%

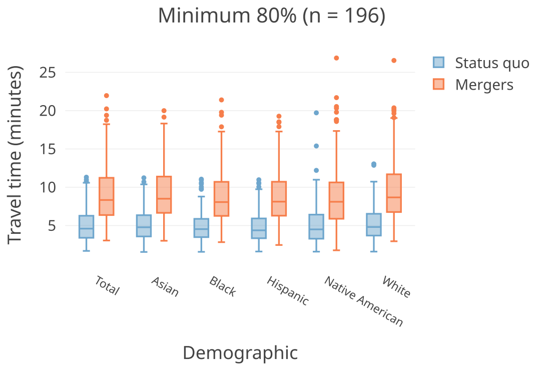

(b) Minimum 80%

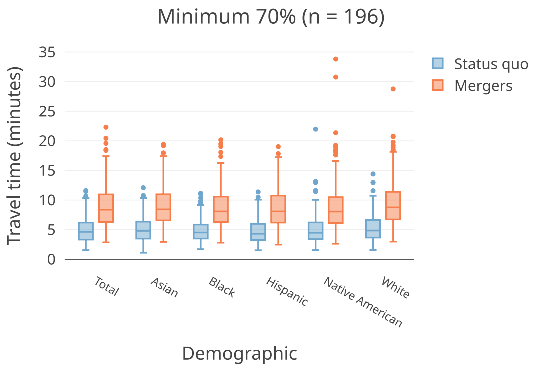

(c) Minimum 70%

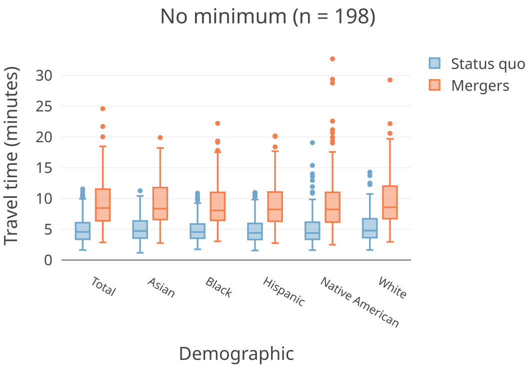

(d) No minimum

Figure S4: Increase in average travel times for students who would switch schools, across demographics, for different minimum enrollment constraints. Sample sizes of less than 200 are due to the failure of the algorithm to find feasible mergers.

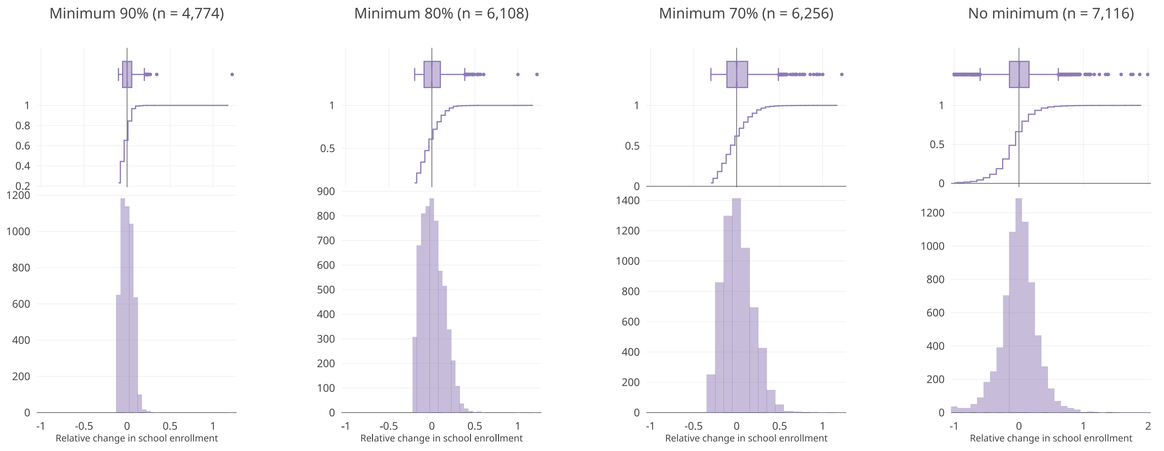

(a) Minimum 90% (b) Minimum 80% (c) Minimum 70% (d) No minimum

Figure S5: Relative changes in school enrollment, 30 bins. Looser constraints on the minimum school enrollment generally affords a greater number of schools to merge, and no minimum enrollment constraint allows for simulating school closures. The top chart shows a cumulative density function; the bottom represents the data as a histogram.

minimum constraint on enrollment (Fig. S5).

Some schools appear on the high end of the cumulative distributions, i.e., their relative enrollment changes of 100% or greater. This occurs for schools with an existing low enrollment, yet historically higher capacity (enrollment). An example is Peeler Open Elementary in Guilford County Schools, which consistently begins with a population of 18 and receives between 22 (×1.22 relative change) and 138 (×7.6 relative change) students depending on the simulation. Schools with relative changes of 2.00 or greater are not included in the Figure S5 plots, for ease of reference.

S1.c.a School closures

Setting the fraction to 0.0 allows the algorithm to propose school closures if such closures would advance the primary objective of reducing racial/ethnic segregation across the district's schools. In these scenarios, one or more neighboring schools would enroll the students previously attending the now-closed school. The distribution of the relative changes in school enrollment for this simulation has a small accumulation at –1.00, i.e., school closure. Of the 7,116 resulting clustered schools, 24 were closures, and 312 schools experienced halving in enrollment or greater. Of the 24, the median pre-merger enrollment is 217, less than the median pre-merger enrollment for all clustered schools, 472.5.

S1.d Inter-district mergers

Inter-district results are summarized in Figure S6.

The median status quo dissimilarity score for the inter-district simulations is 0.49. This score is greater than the dissimilarity score for the primary simulations (0.41) due to a greater prevalence of segregation across district lines (1, 2). The relative decrease in dissimilarity is –12.2%, which is less in magnitude than the intra-district results presented in the main text (–19.5%)—though, importantly, both measures operate over different denominators (inter-district simulations include all of the schools in a given focal district and its adjacent districts; intra-district simulations only include schools in the given focal district). We estimate inter-district mergers would also

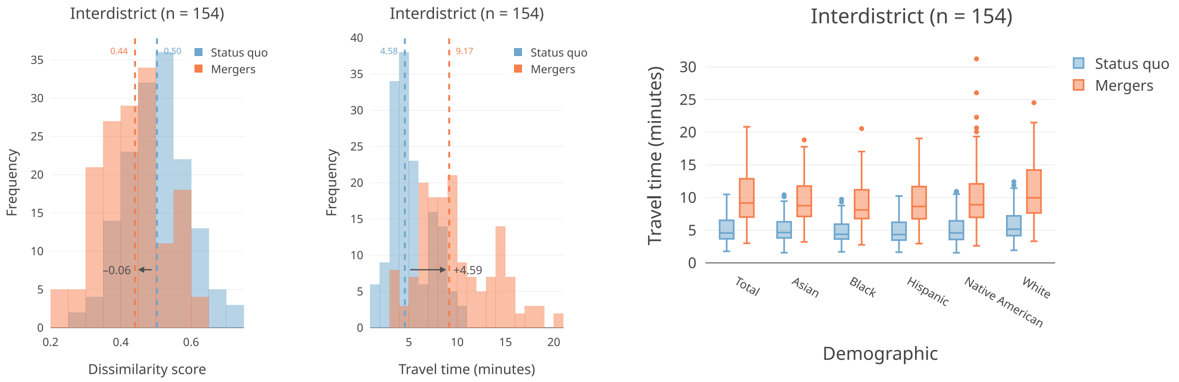

(a) Dissimilarity score (b) Travel times (c) Travel times across demographic

Figure S6: Impact on integration results when allowing for mergers between districts.

increase travel times by nearly 4.5 minutes each way, on average, for students involved in a pairing/tripling—slightly higher, as expected, than the intra-district setting.

If most of the segregation is due to schools that exist on the borders of the neighboring districts, then we might expect a larger decrease by simulating mergers between border schools; the relatively small expected decrease in segregation of 12%, then, may suggest that a large amount of between-district segregation is likely due to schools that are not on such borders.

S2 Data and code release

All code and data required to replicate the main and supplementary results of the paper can be found here: `https://github.com/Plural-Connections/public-school-mergers`.

References

[1] Jill Barshay. 2024. Proof points: 5 takeaways about segregation 70 years after the brown decision.

[2] Jeremy E. Fiel. 2013. Decomposing School Resegregation: Social Closure, Racial Imbalance, and Racial Isolation. *American Sociological Review*, 78(5).

[3] Nabeel Gillani, Doug Beeferman, Christine Vega-Pourheydarian, Cassandra Overney, Pascal Van Hentenryck, and Deb Roy. 2023. Redrawing attendance boundaries to promote racial and ethnic diversity in elementary schools. *Educational Researcher*, page 0013189X231170858.

[4] Hugh Macartney and John D. Singleton. 2018. School boards and student segregation. *Journal of Public Economics*, 164:165–182.

[5] Parag A. Pathak and Peng Shi. 2017. How Well Do Structural Demand Models Work? Counterfactual Predictions in School Choice. *NBER Working Paper No. 24017*.
